# Exploring cervicovaginal microbiome differences between single and multiple endometrial polyps: implications for non-invasive classification

**DOI:** 10.1128/msystems.00023-25

**Published:** 2025-09-15

**Authors:** Tianshu Sun, Qingyue Zheng, Roujie Huang, Leyan Yang, Zimo Liu, Zhibo Zhang, Xudong Liu, Hua Yang, Xiaochuan Li, Jiali Tong, Lan Zhu

**Affiliations:** 1 Clinical Biobank, Center for Biomedical Technology, National Science and Technology Key Infrastructure on Translational Medicine, Institute of Clinical Medicine, State Key Laboratory of Common Mechanism Research for Major Diseases, State Key Laboratory of Complex, Severe, and Rare Diseases, Peking Union Medical College Hospital, Chinese Academy of Medical Sciences and Peking Union Medical College 12501, Beijing, China; 2 Department of Obstetrics and Gynecology, National Clinical Research Center for Obstetric & Gynecologic Diseases, State Key Laboratory of Common Mechanism Research for Major Diseases, State Key Laboratory of Complex, Severe, and Rare Diseases, Peking Union Medical College Hospital, Academy of Medical Sciences and Peking Union Medical College 12501, Beijing, China; 3 Laboratory Animal Research Facility, National Infrastructures for Translational Medicine, Institute of Clinical Medicine, State Key Laboratory of Common Mechanism Research for Major Diseases, State Key Laboratory of Complex, Severe, and Rare Diseases, Peking Union Medical College Hospital, Chinese Academy of Medical Science and Peking Union Medical College 12501, Beijing, China; Southern Medical University, Guangzhou, Guandong, China

**Keywords:** endometrial polyps, reproductive tract microbiome, metagenomic sequencing, biomarker, random forest model

## Abstract

Single and multiple endometrial polyps (EP) are common gynecological conditions with differing recurrence rates, influencing clinical treatment decisions. This study aimed to characterize the reproductive tract microbiome in both subtypes to support the development of methods for the non-invasive categorization of EPs. Using metagenomic sequencing, we analyzed vaginal and cervical samples from 27 reproductive-aged patients with single EP and 22 with multiple EP. Compared with controls and multiple EP cases, single EP vaginal and cervical samples exhibited a lower percentage of community state types (CST) I and II. *Sneathia amnii* was identified as a characteristic species in both the vagina (*P* = 0.0051) and cervix (*P* = 0.0398) of single EP patients compared with controls. *Mesorhizobium* sp. (vaginal *P* = 0.0110, cervical *P* = 0.0210), *Acinetobacter baumannii* (vaginal *P* = 8.0 × 10^−5^, cervical *P* = 0.0314), and *Pasteurella multocida* (vaginal *P* = 0.0173, cervical *P* = 0.0210) were enriched in single EP compared with multiple EP. Kyoto Encyclopedia of Genes and Genomes (KEGG) enrichment analysis of vaginal microbiome revealed unique pathways in single EP, including aminoacyl-tRNA biosynthesis, pantothenate and coenzyme A (CoA) synthesis, pyrimidine metabolism, glycolysis/gluconeogenesis, and biosynthesis of phenylalanine, tyrosine, and tryptophan. Using a random forest model, we further selected microbiota and clinical parameters to differentiate single and multiple EPs, thus achieving an area under curve (AUC) of 0.861. Our findings characterized the composition of the cervicovaginal microbiota of single and multiple EPs and proposed biomarkers for their non-invasive classification based on a random forest model.

**IMPORTANCE:**

The prevalence rate of endometrial polyps (EPs), a common gynecological condition, varies between 7.8% and 34.9%. Multiple EPs are associated with higher recurrence rates and chronic endometritis than single EPs and thus require more aggressive clinical interventions. However, only laparoscopic surgery can accurately identify single and multiple polyps. Non-invasive adjunctive diagnostic methods can aid in altering surgical indications preoperatively. Using metagenomic sequencing, we thoroughly analyzed the vaginal and cervical samples of 27 single EP and 22 multiple EP patients of reproductive age. We then identified distinct microbial patterns in the single and multiple samples, which were crucial for understanding EP pathogenesis and its association with gynecological health. Using a random forest model, key bacterial taxa that differentiate single and multiple EPs were identified with high accuracy. These could potentially serve as non-invasive diagnostic biomarkers. This research delineates the cervicovaginal microbiome of the reproductive tract in EP patients, offering a basis for developing non-invasive diagnostic tools and personalized treatment strategies.

## INTRODUCTION

Endometrial polyps (EPs) are a common gynecological disorder with an incidence rate of 7.8%–34.9% (1, 2). They are characterized by the overgrowth of endometrial tissues, which often triggers abnormal uterine bleeding and may even contribute to infertility. Multiple EPs, defined in our study as the presence of three or more polyps, are significantly more recurrent than single EPs (3, 4) and thus require more intensive clinical interventions (5). Accurate differentiation between single and multiple polyps impacts clinical decision-making and offers valuable insights into their unique pathogenic mechanisms. Hence, a supplementary diagnostic approach must be developed. This is particularly important when ultrasound findings are inconclusive or further polyp characterization is required. In reproductive-aged women, multiple EPs are positively correlated with chronic endometritis, suggesting a role for chronic inflammation in their development (6). According to molecular-level studies, expression levels of the estrogen receptor, vascular endothelial growth factor, and transforming growth factor β1 are elevated in multiple EPs compared with single EPs (7). Although the exact pathogenesis of both polyp types remains elusive, evidence suggests they could be categorized into two subtypes (5).

Mounting evidence has revealed that the reproductive tract microbiome in females is strongly correlated with their overall health status. Alterations in the microbiome composition often reflect disease states. For example, fluctuations in *Lactobacillus* abundance have been closely linked to numerous common gynecological disorders. Reduced vaginal *Lactobacillus* abundance is linked to uterine fibroids (8), endometriosis (9, 10), and endometrial cancer (11). Conversely, a proliferation of vaginal *Lactobacillus* may be related to adenomyosis (12) and endometrial hyperplasia (13). These findings underscore the significance of *Lactobacillus* as a dominant microbiota in the female reproductive tract, with substantial changes in its abundance predicting gynecological diseases. Moreover, the abundance of bacteria, such as *Firmicutes* (11, 14–18) and *Streptococcus* (11, 16, 19, 20), increases in various gynecological diseases, which indicates their possible detrimental effects on female reproductive health. Monitoring the abundance of these bacteria could thus offer valuable disease risk-related information.

EP pathogenesis involves inflammation and infection, with microbiota potentially contributing by modulating local immunity, mediating inflammatory responses, promoting the growth of pathogens, and influencing cell proliferation and apoptosis (14, 21, 22). Studies on the EP microbiome have mainly assessed microbial diversity and variations at the phylum and genus levels. Fang et al. found reduced *Pseudomonas* and increased *Lactobacillus*, *Gardnerella*, *Bifidobacterium*, *Streptococcus,* and *Atopobium* in the uterine microbiota of EP patients (14), though the small sample size (*n* = 10 per group) limits generalizability. Liang et al. reported significantly increased *Bacteroides* and decreased *Proteobacteria* in the vagina, cervix, and uterine cavity of EP patients compared with controls (16). Although Liang et al.’s (16) study involved a large sample size and comprehensive sampling sites, all participants were infertile, introducing possible hormonal confounding. Given the close associations between EPs, inflammation, and infection, the cervicovaginal microbiota likely plays a pivotal role in their pathogenesis. Therefore, investigating the microbiome differences in EP patients is of great importance.

Here, we recruited a more diverse and representative patient cohort and applied metagenomic sequencing to explore species-level changes in the cervicovaginal microbiota in both single and multiple EP cases. Our aim was to deepen our understanding of EP pathogenesis and identify non-invasive microbiological biomarkers for its diagnosis.

## MATERIALS AND METHODS

### Participant enrollment

Preoperative reproductive tract microbiome samples were collected from EP patients at the Obstetrics and Gynecology Department of Peking Union Medical College Hospital (PUMCH) between December 2022 and April 2023. Reproductive-age women without any reproductive tract organic lesions, who attended the same department concurrently, were enrolled in the control group. The inclusion criteria of the patients with EP were a clinical diagnosis of EP based on symptoms and imaging, or intraoperative findings and post-operative pathological examination of resected tissue; age 18–45 years. For the control group, the criteria were no detected reproductive tract organic pathology based on clinical manifestations, physical examination by specialists, examination tests, endoscopy, and surgery; age 18–45 years. Patients were excluded if they had reproductive tract or systemic infectious diseases (e.g., trichomonas vaginitis, pelvic inflammatory disease, human papillomavirus infection, hepatitis B virus, and syphilis infection); reproductive system malignant tumors; autoimmune diseases; antibiotic use within 14 days before sampling; sexual intercourse, vaginal douching, or medication used within 7 days before sampling; estrogen or tamoxifen therapy undertaken within the past month; chronic diseases (such as hypertension or diabetes).

Reproductive tract microbiome samples were collected from 48 cases of EP, including 27 cases of single EP, 22 cases of multiple EPs, and 27 cases of controls (Table 1). No statistically significant differences were detected among the groups in terms of age, body mass index (BMI), polyp size, and endometrial thickness.

**TABLE 1:** The clinical features of the women participants in the study

Clinical characteristics	Single EP	Multiple EPs	Control
Age (years)	37.59 ± 5.918	37.68 ± 5.801	30.63 ± 5.300
Body mass index (BMI) (kg/m^2^)	21.20 ± 2.766	23.43 ± 3.720	21.61 ± 3.554
Size of polyps (cm)	1.172 ± 0.387	1.585 ± 1.591	Not applicable
Endometrial thickness (cm)	0.971 ± 0.324	0.996 ± 0.376	0.857 ± 0.441
Abnormal uterine bleeding	16/27 (59.3%)	4/22 (18.2%)	7/27 (25.9%)
History of progesterone treatment	8/27 (29.6%)	3/22 (13.6%)	2/27 (7.41%)

### Sample collection and sequencing

All samples were collected during the endometrial proliferative phase. To characterize the vaginal or cervical microbial community, sterile cotton swabs were carefully placed into the vaginal or cervical canal, rotated to dislodge adherent microorganisms, and then immediately placed into sterile cryovials, sealed, and stored at −80°C. Total nucleic acids extraction was conducted with the FastPure Bacteria DNA Isolation Mini Kit (Vazyme), followed by sequencing library preparation by using the TruSeq DNA PCR-Free Sample Preparation Kit (Illumina). Library quality was performed on the Qubit 2.0 Fluorometer (Thermo Scientific), and sequencing was performed on an Illumina NovaSeq platform.

### Data analysis

Data quality control and host sequence removal were performed using the KneadData software. Initially, FastQC software was applied to inspect the key quality parameters of raw sequencing data. Subsequently, Trimmomatic software was applied to trim adapters, eliminate low-quality bases, and filter out sequences of insufficient quality. Next, alignment against the human reference genome sequence (GRCh38) was conducted using Bowtie2 to exclude host-derived sequences from the data set. Finally, a post-alignment quality check was performed using FastQC software to confirm adherence to the analysis standards. Taxonomic annotation was performed using MetaPhlAn 3.0 software to swiftly determine the composition of microbial communities (including bacteria, archaea, eukaryotes, and viruses). Post-quality-controlled sequences were aligned to marker gene databases using Bowtie2 (Version: 2.4.2), facilitating rapid and comprehensive species identification.

### Model building

The classification ability of the vaginal microbiota in predicting EPs was assessed by applying the random forest algorithm using the R package randomForest (4.7-1.1) (23). To address sample size imbalance, oversampling was performed and followed by equitable distribution of the samples. The data set was then split into training (70%) and testing (30%) subsets. For model training, the training subset was used with hyperparameter tuning via the caret package (24). The optimal 10-fold cross-validation model parameters were mtry =10 and ntree =500. Random forest analysis predicted outcome variables (single EP or multiple EPs). Variable significance was assessed using *P* values from the rfPermute package (v2.5.2) (25). Receiver operating characteristic (ROC) curves (pROC package) were used to validate the model on the testing subset.

In the first random forest analysis, all taxa’s relative abundances and clinical parameters were included. Significant variables (top 20 mean decrease accuracy or mean decrease Gini scores) were applied in a second random forest analysis with parameters mtry =2 and ntree =1000. Variable importance was ranked, and the model performance was evaluated based on the ROC curves and AUC values. For single EP and multiple EPs analysis, the parameters were mtry =10, ntree =500. For control, single EP, and multiple EPs analysis, the parameters were mtry =14, ntree =100. The DeLong method was used to calculate the ROC curve confidence interval. SHapley Additive exPlanation (SHAP) values were analyzed using the R package fastshap (v0.1.1) after Min-Max [0, 1] scaling. The top-10 SHAP mean value illustrates each taxon’s contribution and direction to the predicted label.

### Statistical analyses

The clinical information was analyzed using SPSS 25.0 software. Analysis of alpha diversity was leveraged using Wilcoxon’s test. Principal coordinates analysis (PCoA) was implemented via permutational multivariate analysis of variance (Adonis) test. Community state type (CST) and linear discriminant analysis effect size methods were performed to test the difference in the species composition. HUMAN 3.04 and the UniRef90 reference database were used for KEGG functional annotations. The differences analysis was conducted using Welch’s *t*-test.

## RESULTS

### Vaginal and cervical microbiota composition and variability in EPs

**Fig 1:**
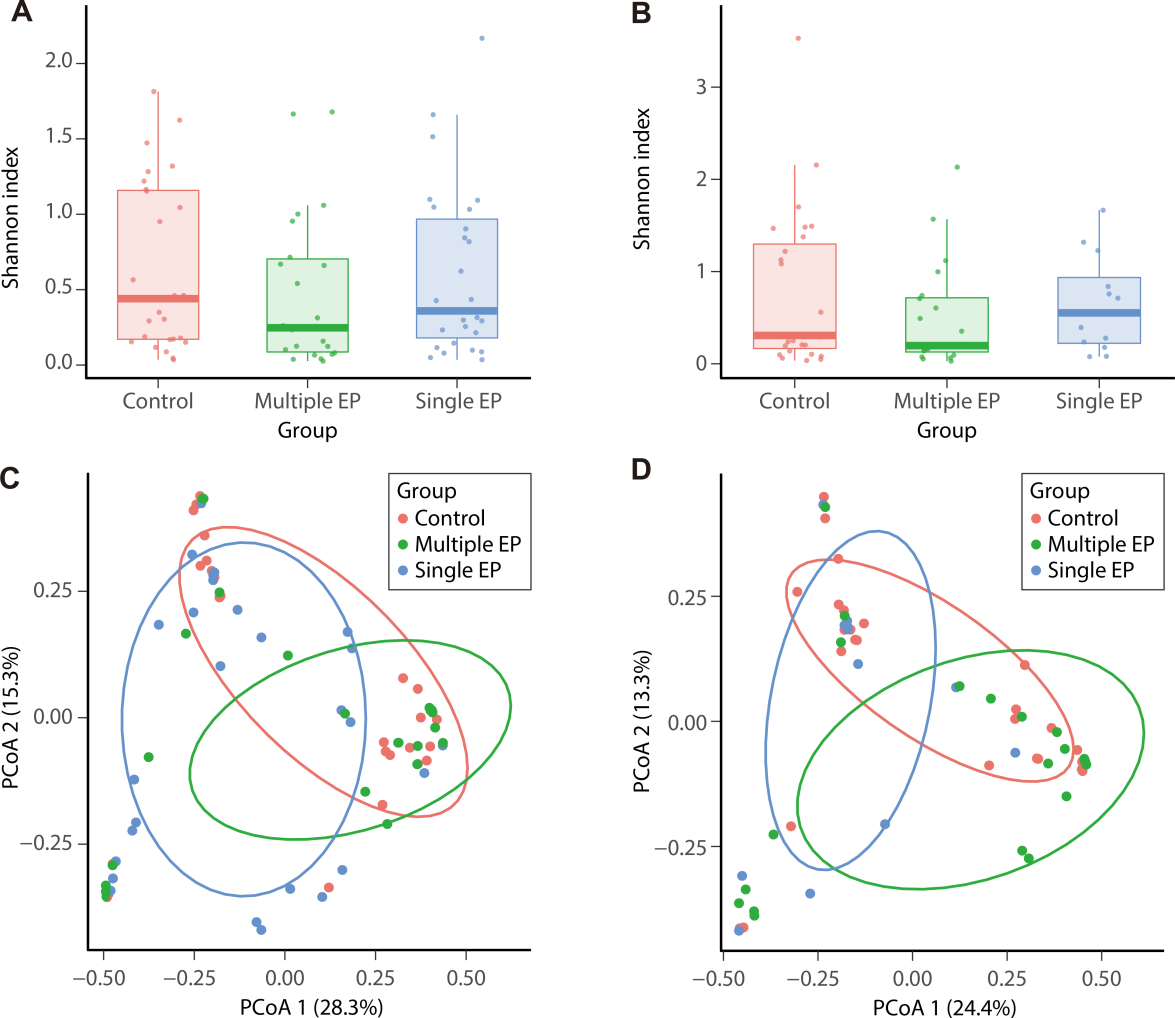
Species diversity of single EPs, multiple EPs, and controls. (**A**) Vaginal α-diversity; (**B**) cervical α-diversity. Horizontal axis-group; vertical axis-Shannon index. (**C**) PCoA analysis of vaginal species; (**D**) PCoA analysis of cervical species. Horizontal axis-PCoA 1; vertical axis-PCoA 2. Single EP group (*n* = 27), multiple EP group (*n* = 22), and control group (*n* = 27).

We first assessed the α-diversity of the microbiota across different disease states using the Shannon index, which measures species diversity within samples. The Shannon index for both the vagina and cervix exhibited an increasing trend from multiple EPs (vagina 0.481, cervix 0.502) to single EPs (vagina 0.601, cervix 0.646) and then to controls (vagina 0.638, cervix 0.744); these differences were not statistically significant (Fig. 1A and B). To further explore microbiota structure, we performed PCoA based on species abundance. The PCoA plots and Bray-Curtis distances (Table S1) unveiled that β-diversity and microbiota structures in controls, single EPs, and multiple EPs were similar for both the vagina and cervix (Fig. 1C and D).

**Fig 2:**
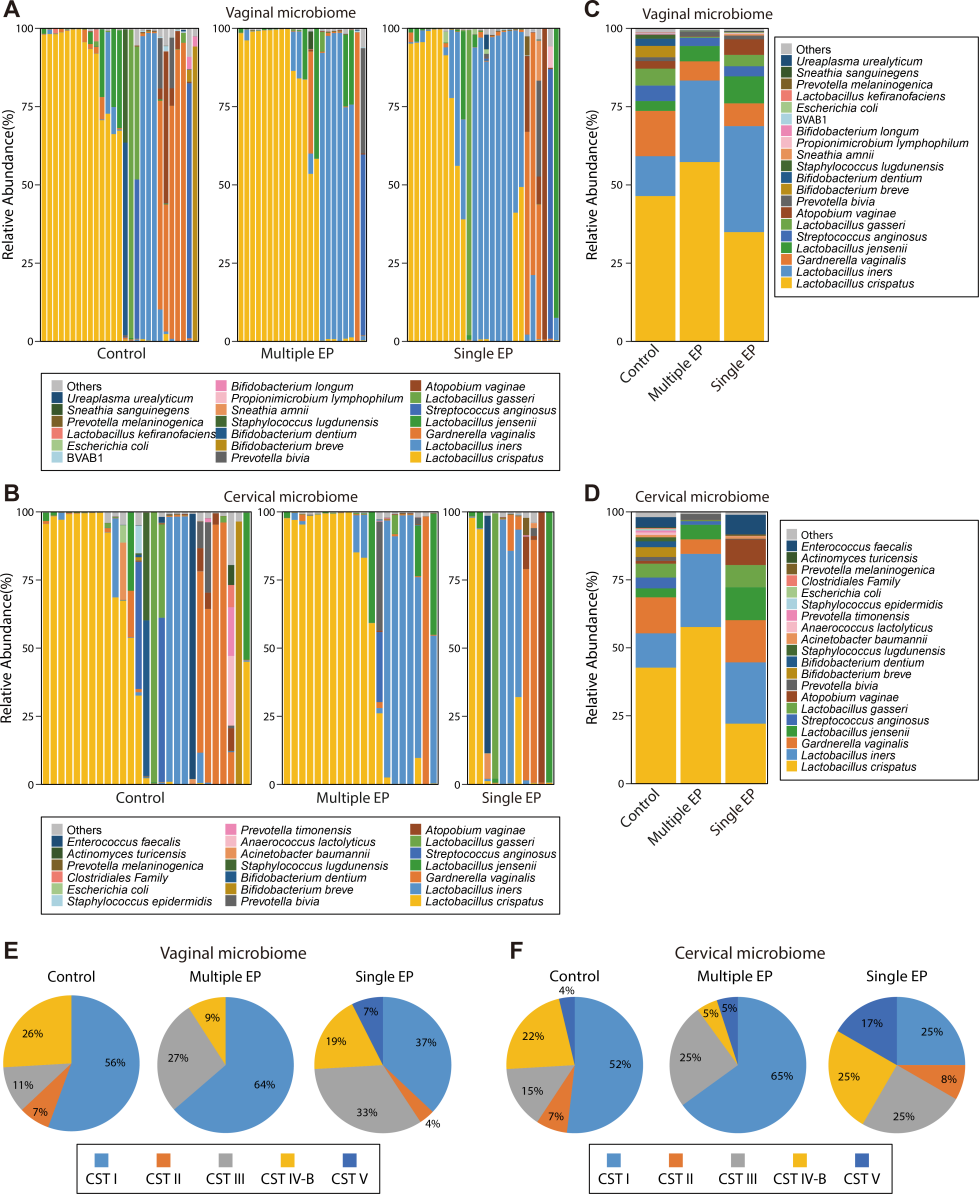
Relative abundance of species in single EPs, multiple EPs, and controls. (**A**) Histogram of relative abundance at the vaginal species level; horizontal axis-samples; vertical axis-relative proportions; see legend on the right for the species categories corresponding to each color block. (**B**) Histogram of relative abundance at the cervical species level. (**C**) Histogram of vaginal microbiota mean relative abundance at the vaginal species level. (**D**) Histogram of cervical microbiota mean relative abundance at the species level. (**E**) Pie chart of CST proportions for vaginal samples. (**F**) Pie chart of CST proportions for cervical samples. Single EP group (*n* = 27), multiple EP group (*n* = 22), and control group (*n* = 27).

Then, we selected the top 20 species by relative abundance, grouping the remaining species as “others” to generate a bar chart (Fig. 2; Table S2). Across all groups, *Lactobacillus crispatus*, *Lactobacillus iners*, *Gardnerella vaginalis*, and *Lactobacillus jensenii* were dominant in both the vaginal and cervical microbiomes, comprising 82%–99% of the total microbial content in each group. A distinct microbial pattern related to polyp occurrence emerged. *L. crispatus* was less prevalent in the vagina and cervix of single EPs [relative abundance: 0.3493 (v)/0.2209 (c)] compared with multiple EPs [0.5732 (v)/0.5763 (c)] and controls [0.4645 (v)/0.4269 (c)] (Fig. S1). By contrast, *L. iners* and *L. jensenii* were elevated in both single and multiple EPs. The relative abundance for *L. iners* was 0.1273 (v)/0.1262 (c) in controls, 0.3386 (v)/0.2255 (c) in single EPs, and 0.2604 (v)/0.2693 (c) in multiple EPs. For *L. jensenii*, the values were 0.0317 (v)/0.0329 (c) in controls, 0.0862 (v)/0.1210 (c) in single EPs, and 0.0490 (v)/0.0538 (c) in multiple EPs. Furthermore, the prevalence of *Lactobacillus kefiranofaciens* was even lower (relative abundance: 0.0020 (v)/0.0020 (c) in controls, 0.0002 (v)/0.0001 (c) in single EPs, and 0.0001 (v)/0.0001 (c) in multiple EPs). These findings suggest that *Lactobacillus* distribution in the vagina and cervix might vary with polyp development, providing insight into microbial dynamics in female reproductive health and diseases. In addition, *Atopobium vaginae*, previously reported to be associated with endometrial cancer (26), was elevated 6.2-fold (vagina) and 21.5-fold (cervix) in single EPs compared with controls. Further studies are warranted to clarify the possible associations between these microbial taxa and the pathophysiology mechanisms underlying EPs.

In gynecological microbiome research, the classification of vaginal microbial communities into distinct categories, known as CST, has been widely used for understanding complex interactions between microbial composition and vaginal health. Our investigation, based on CST, has unveiled that CST I is predominant in both vaginal and cervical compartments of controls and single and multiple EP patients. CST I is defined by the predominance of *L. crispatus*, a species widely known for maintaining the vaginal ecosystem’s homeostasis. In single EPs (25.00% in the vagina, 20.00% in the cervix) and multiple EPs (21.43% in the vagina, 20.00% in the cervix), there was a higher proportion of CST III, primarily characterized by *L. iners* and associated with reduced microbiota stability (27), compared with controls (10.00% in the vagina, 12.90% in the cervix). However, these differences were not statistically significant (*P* > 0.05). The CST analysis underscores the vaginal health-preserving significance of *L. crispatus* across all groups. Meanwhile, the higher occurrence of CST III in EP groups implies a potential association between vaginal dysbiosis and EPs. These observations advocate for a more nuanced appreciation of the complex role of the vaginal microbiome in gynecological disorders and the need for targeted therapeutic interventions for rectifying microbial imbalances.

**Fig 3:**
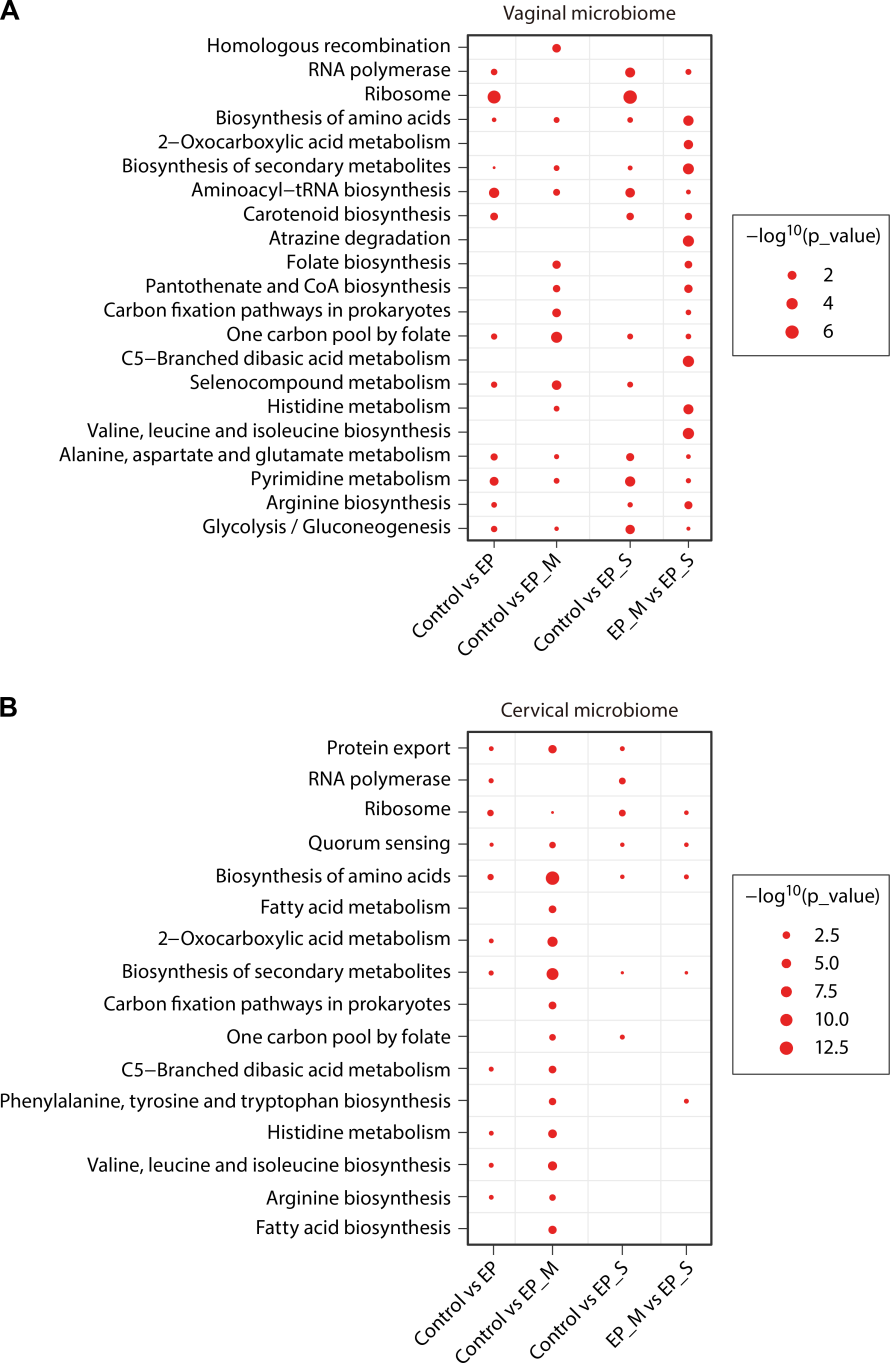
KEGG pathway enrichment of DEGs across single EPs, multiple EPs, and controls. KEGG enrichment analysis of the identified DEGs between EPs and control groups, single EP and control groups, multiple EP and control groups, single and multiple EP groups for vaginal samples (**A**) and cervical samples (**B**). Single EP group (*n* = 27), multiple EP group (*n* = 22), and control group (*n* = 27).

KEGG enrichment analysis of differentially expressed genes (DEGs) in the vaginal microbiota indicated that metabolic pathways were predominant within the KEGG pathway annotations of the microbiome (Table S3). Significant differences between single and multiple EPs included aminoacyl-tRNA biosynthesis, pantothenate and coenzyme A (CoA) synthesis, pyrimidine metabolism, and glycolysis/gluconeogenesis (Fig. 3A). In cervical samples, phenylalanine, tyrosine, and tryptophan biosynthesis was enriched in multiple EPs compared with single EPs (Fig. 3B). These results suggest distinct metabolic alterations in vaginal microbes associated with EP pathologies and propose novel avenues for clinical treatment targeting specific metabolic pathways.

### Distinct microbial signatures in the cervical and vaginal microbiome of EPs

**Fig 4:**
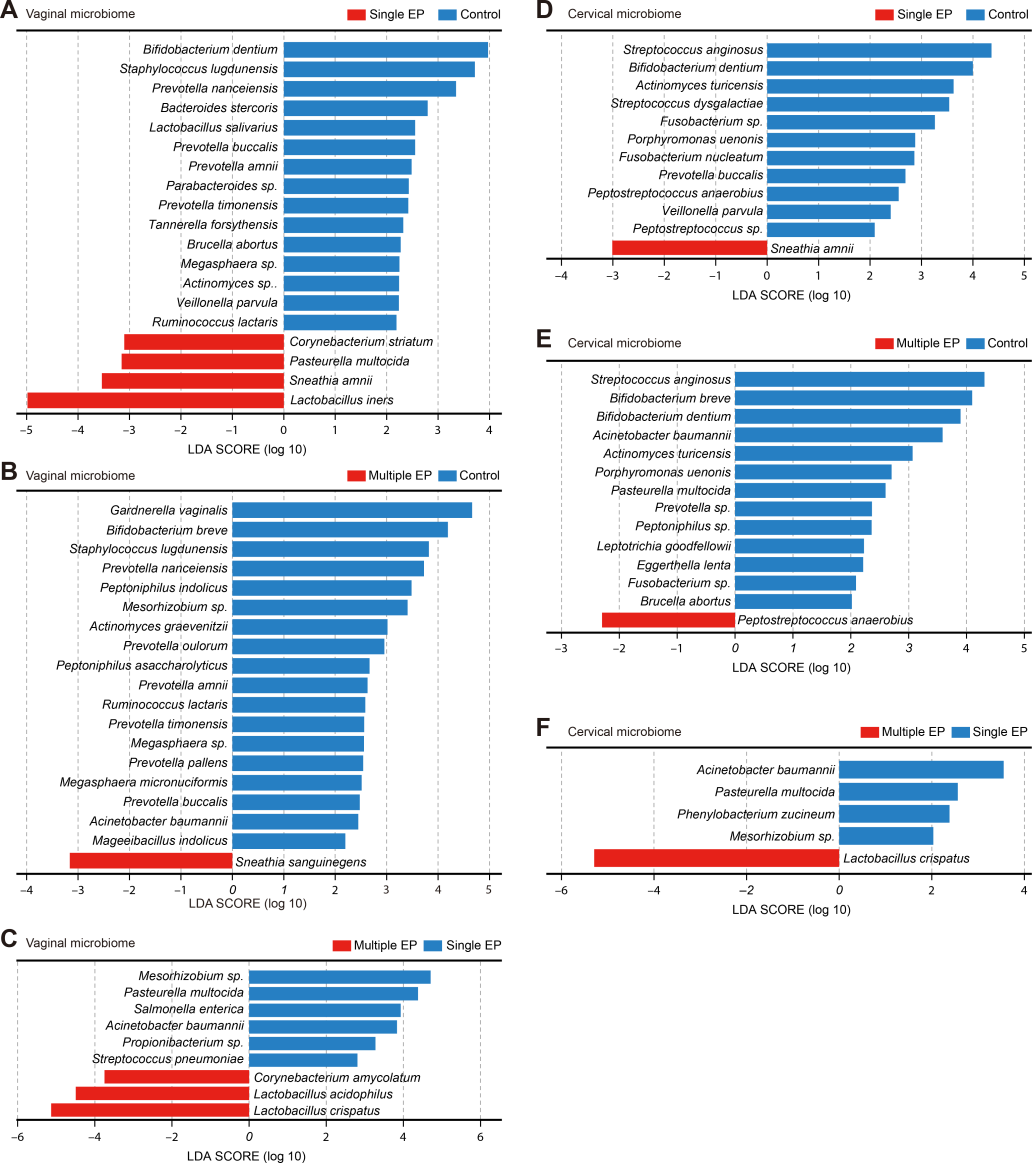
Differential species for single EP, multiple EP, and control group. (**A**) Vaginal differential species between the single EP and control groups. (**B**) Vaginal differential species between multiple EP and control groups. (**C**) Vaginal differential species between single EP and multiple EP groups. (**D**) Cervical differential species between the single EP and control groups. (**E**) Cervical differential species between the multiple EP and control groups. (**F**) Cervical differential species between the single EP and multiple EP groups. Horizontal axis-log LDA score; vertical axis-species; see legend above for groups corresponding to each color block. Single EP group (*n* = 27), multiple EP group (*n* = 22), and control group (*n* = 27). Microorganisms meeting the criteria of *P* < 0.05 in the rank-sum test for differences in species abundance between groups and the absolute value of the LDA score >2 were considered as differential species.

Utilizing the rank-sum test and linear discriminant analysis (LDA) scoring, we identified distinct microbial signatures for each group, thereby providing major insights into the cervical and vaginal characteristic species of EPs (Fig. 4; Table S4).

A stark difference in microbial composition was noted between the single EP and control groups. In the vaginal microbiome of single EPs, *Sneathia amnii* and three other bacterial species were predominant as characteristic species. By contrast, the control group’s vaginal microbiota involved 15 bacterial species, including *Prevotella buccalis*, *Staphylococcus lugdunensis,* and *Bifidobacterium dentium*. In the cervical region of single EP patients, *S. amnii* (*P* < 0.05 and an absolute LDA score of >3) was a distinctive species. Meanwhile, the control group’s cervical microbiome had 11 bacterial species, including *B. dentium*.

On comparison, we found that *Sneathia sanguinegens* was a characteristic vaginal species in multiple EPs (*P* < 0.05 and an absolute LDA score of >3), whereas 18 bacterial species, such as *P. amnii* and *G. vaginalis*, were characteristic of the control group. *Peptostreptococcus anaerobius* was the characteristic cervical species in multiple EPs, and 13 bacteria, including *Streptococcus anginosus*, were the characteristic cervical species in the controls.

A comparison between single and multiple EP groups unveiled that in the vaginal microbiome, *L. crispatus*, *Lactobacillus acidophilus*, and *Corynebacterium amycolatum* were characteristic of multiple EPs, with an absolute LDA score of >3. Conversely, *Propionibacterium* sp., *Mesorhizobium* sp., *Streptococcus pneumoniae*, *Acinetobacter baumannii*, *Salmonella enterica*, and *Pasteurella multocida* were characteristic vaginal species in single EPs. In cervical samples, *L. crispatus* was characteristic of multiple EPs, with an absolute LDA score of >5. *Phenylobacterium zucineum*, *Mesorhizobium* sp., *P. multocida*, and *A. baumannii* were characteristic of single EPs.

The differential abundance of specific microbial species across varying EP conditions highlights the microbiome’s intricate interplay with EPs. *Sneathia* species, consistently identified in single EPs, and *L. crispatus,* distinctively present in multiple EP cases, may play a role in the pathophysiology of different EP subtypes. These findings indicate that the mechanistic links between the microbiome and EP, which could lead to targeted treatments and a better understanding of EP etiology, must be explored further.

### Identification of single and multiple EPs based on vaginal microbiota and clinical parameters

**Fig 5:**
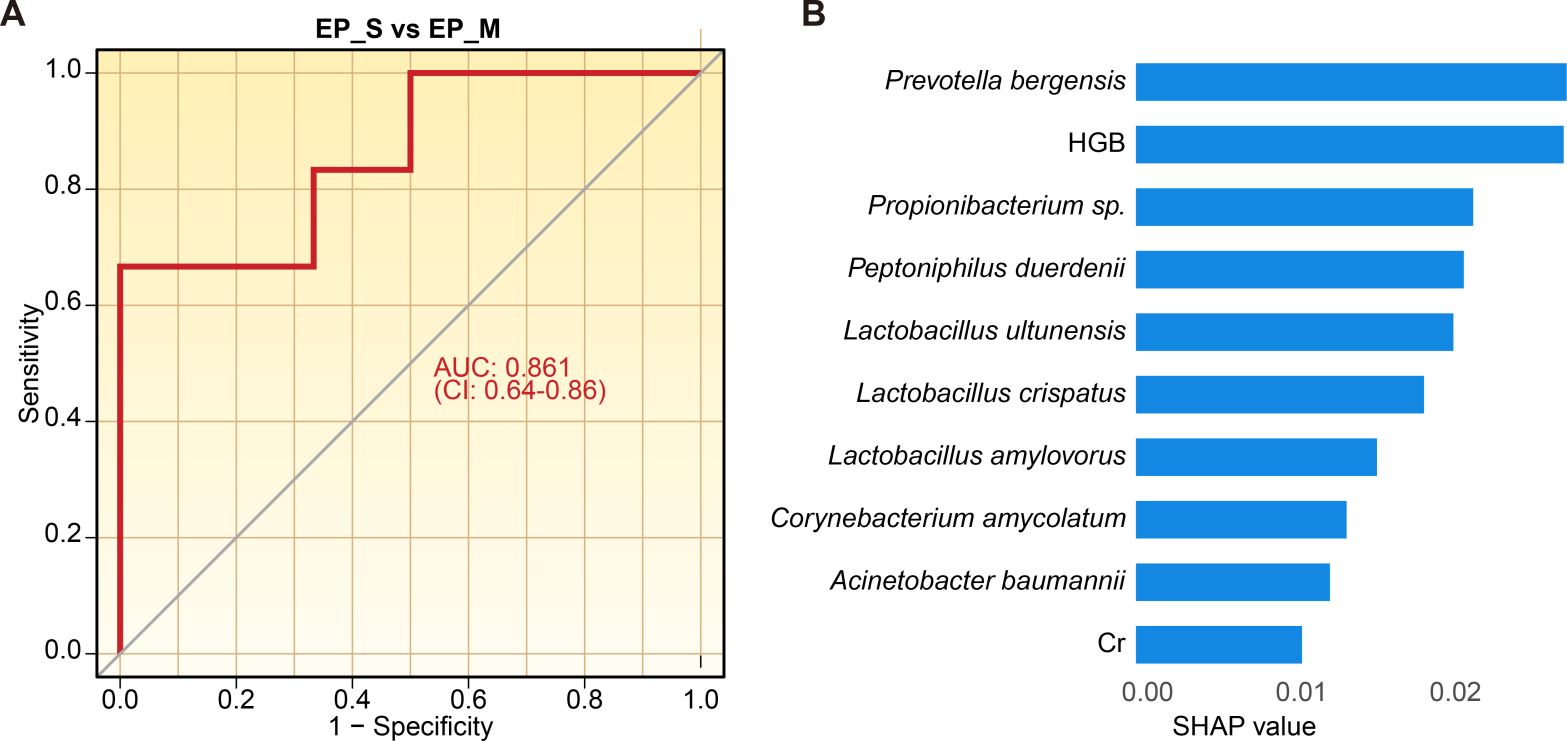
The ROC curve of the random forest model using 20 selected variables to distinguish between single EP and multiple EP. (**A**) ROC curve of single EP vs multiple EP. (**B**) SHAP value of the TOP 10 taxa in the random forest model of single EP vs multiple EP.

As vaginal microbiota composition varied markedly between the single EP and multiple EP groups, we evaluated the potential of vaginal microbiota to distinguish between these subtypes. Initially, relative abundances of all taxa and essential clinical parameters (Table S5) were incorporated into the first random forest analysis, and an out-of-bag (OOB) estimate of error rate of 16.67% was achieved. Subsequently, the top 20 variables ranked by mean decrease accuracy and mean decrease Gini, which were statistically significant in this analysis, were selected for a second random forest analysis. This refined model demonstrated an improved predictive accuracy, with a reduced OOB estimate of error rate of 3.33%. The second model, using these selected variables, had an AUC of 0.861 (Fig. 5A).

Our findings highlighted specific taxa, namely *L. crispatus*, *Propionibacterium* sp., *A. baumannii,* and *Enterococcus faecium*, as pivotal in differentiating single EPs from multiple EPs. These results were validated through Spearman’s relative analysis or random forest modeling (Table 2). Our results underscore the vaginal microbiota’s role as a sensitive and specific marker for distinguishing single EPs from multiple EPs.

**TABLE 2:** Spearman’s relative analysis and random forest modeling of the 20 selected variables for distinguishing single and multiple EPs

Variables	Mean decrease accuracy	*P* value of mean decrease accuracy	Mean decrease Gini	*P* value of mean decrease Gini	R value of Spearman	*P* value of Spearman
AST	5.3831	0.0099	0.5851	0.0099	−0.20168	0.2382
*Lactobacillus crispatus*	5.1673	0.0099	0.5875	0.0099	−0.42577	0.0096
*Propionibacterium* sp.	4.7575	0.0198	0.4938	0.0495	0.489939	0.0024
Fbg	4.3611	0.0297	0.3719	0.1287	−0.34443	0.0397
PT	4.1636	0.0396	0.3914	0.0396	0.18792	0.2724
*Atopobium vaginae*	4.1081	0.0198	0.1911	0.3762	0.258211	0.1284
*Gardnerella vaginalis*	4.0808	0.0198	0.3005	0.1584	0.306442	0.0691
*Acinetobacter baumannii*	4.0564	0.0099	0.5681	0.0099	0.566342	0.0003
ALT	3.9427	0.0198	0.2735	0.1485	−0.28863	0.0878
*Lactobacillus fermentum*	3.6839	0.0099	0.2212	0.0792	−0.12299	0.4749
*Prevotella bergensis*	3.6756	0.0297	0.2601	0.0594	−0.18059	0.2919
*Streptococcus agalactiae*	3.6087	0.0297	0.2217	0.0693	0.250555	0.1405
*Enterococcus faecium*	3.5980	0.0297	0.3309	0.0099	0.455786	0.0052
*Corynebacterium amycolatum*	3.3078	0.0099	0.2454	0.0099	−0.32644	0.0520
Urea	3.2830	0.0198	0.1457	0.8713	−0.14917	0.3852
*Lactobacillus johnsonii*	3.2720	0.0297	0.2644	0.1881	0.255081	0.1332
*Peptoniphilus duerdenii*	3.1937	0.0297	0.2226	0.0297	−0.21604	0.2057
Cr	3.0314	0.0297	0.2434	0.2772	−0.19554	0.2531
*Prevotella disiens*	3.0131	0.0198	0.1648	0.3267	−0.15853	0.3558
*Mageeibacillus indolicus*	2.6450	0.0594	0.3578	0.0297	0.32364	0.0542

**Fig 6:**
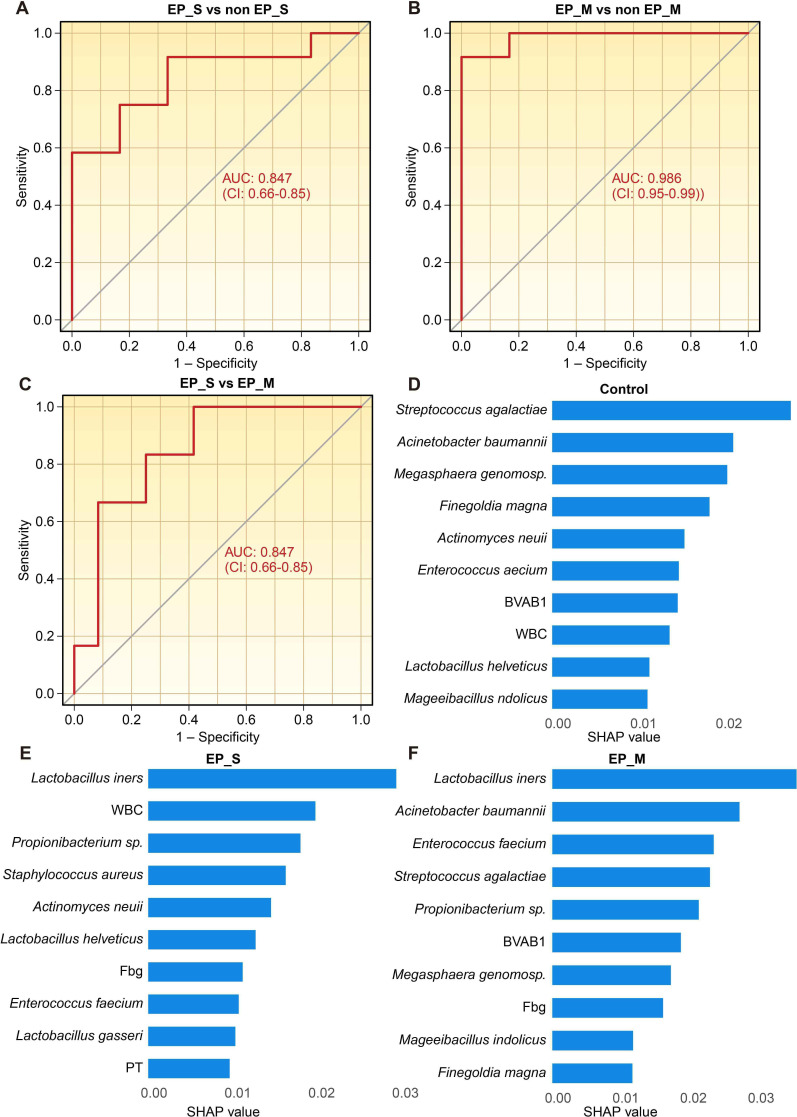
The ROC curve of the random forest model using selected variables among healthy, single EP, and multiple EP. (**A**) Single EP vs non-single EP. (**B**) Multiple EP vs non-multiple EP. (**C**) Single EP vs multiple EP. (**D**) SHAP value of the TOP 10 taxa in the random forest model of Control. (**E**) SHAP value of the TOP 10 taxa in the random forest model of single EP. (**F**) SHAP value of the TOP 10 taxa in the random forest model of multiple EP.

Developing a diagnostic model that can distinguish among healthy individuals, those with single EPs, and those with multiple EPs would hold greater value. Using the same methodology, we further assessed whether vaginal microbiota can be used to differentiate among these three groups. The OOB estimate of error rate was 35.56% and 26.67% in the first random forest analysis and the refined model, respectively. The refined model achieved robust discrimination across all pairwise comparisons (Fig. 6A through C; Table 3): single EP vs non-single EP: AUC = 0.847 (95% CI 0.66–0.85), sensitivity 0.83, and specificity 0.73. Multiple EP vs non-multiple EP: AUC = 0.986 (95% CI 0.95–0.99), sensitivity 0.95, and specificity 0.96. Single EP vs multiple EP: AUC = 0.847 (95% CI 0.66–0.85), sensitivity 0.81, and specificity 0.77. The higher AUC for multiple EP versus non-multiple EP demonstrated the model’s efficiency in identifying multiple EP cases.

**TABLE 3:** Spearman’s relative analysis and random forest modeling of the 14 selected variables for distinguishing among control, single, and multiple EPs

Variables	Mean decrease accuracy	*P* value of mean decrease accuracy	Mean decrease Gini	*P* value of mean decrease Gini
WBC	3.2712	0.0099	0.7095	0.0792
*Streptococcus agalactiae*	3.1075	0.0099	0.9373	0.0099
Fbg	2.4616	0.0297	0.7275	0.0396
*Propionibacterium* sp.	2.3513	0.0198	0.7894	0.0396
*Finegoldia magna*	2.3144	0.0198	0.5671	0.0990
*Lactobacillus iners*	3.4536	0.0099	1.4090	0.0099
*Enterococcus faecium*	2.5897	0.0099	0.8764	0.0099
Urea	2.2755	0.0198	0.7759	0.0594
*Megasphaera_genomo* sp.	2.5926	0.0099	0.7685	0.0198
*Acinetobacter baumannii*	2.6585	0.0099	0.7514	0.0099
BVAB1	1.9730	0.0396	0.6936	0.0693
*Mageeibacillus indolicus*	2.5860	0.0099	0.6901	0.0099
*Lactobacillus coleohominis*	2.3107	0.0297	0.5295	0.1386
*Actinomyces neuii*	2.1101	0.0297	0.5019	0.1584

To enhance clinical interpretability, the random forest analysis was supplemented with SHAP analysis. The 10 most influential taxa of the model’s predictive power were listed (Fig. 5B and 6D through F). In the single vs multiple EP model, the depletion of *L. crispatus* (SHAP value = 0.0187) and the enrichment of *A. baumannii* (SHAP value = 0.0126) and *Propionibacterium* sp. (SHAP value = 0.0219) were the strongest drivers for differentiating simple EP cases from multiple EPs. In the multivariate model among control, single EP, and multiple EP, the abundance of *Lactobacillus ines* in vaginal secretion and the fibrinogen (Fbg) level in blood were the strongest drivers for classifying both single EP and multiple EP from other conditions.

## DISCUSSION

Our research offers an in-depth analysis of vaginal and cervical microbiota in individuals with EPs, providing important insights into microbial diversity and composition across different EP conditions. By comparing single EPs, multiple EPs, and healthy controls, we identified unique microbial signatures and species potentially involved in EP pathophysiology.

Analysis of the relative abundance of microbial species revealed that the vaginal and cervical microbiomes across all groups were predominantly dominated by *L. crispatus*, *L. iners*, *G. vaginalis*, and *L. jensenii*, consistent with previous studies (8, 16). Notably, we observed a significant variation in *Lactobacillus* species distribution in EP cases, reinforcing the possibility of a distinct microbial profile in polyp development. *Atopobium vaginae* was more prevalent in both the vagina and cervix of single EP cases than in controls, suggesting a potential association between specific microbial species and EP formation. Alpha diversity and β diversity analyses showed similar microbial diversity and structures among the control, single EP, and multiple EP groups, in agreement with findings from Liang et al. (16). This suggests that although overall microbial community structures are comparable across different EP conditions, subtle differences may still have clinical relevance.

Using the CST classification method of Ravel et al., studies have shown that the vaginal microbiome of premenopausal women is predominantly CST I (dominated by *L. crispatus*) and CST III (dominated by *L. iners*) (28). In our study, CST I was predominant across all groups; however, both single and multiple EP groups exhibited a higher prevalence of CST III, which is associated with dysbiosis and reduced microbiota stability. This shift toward CST III suggests a potential link between vaginal dysbiosis and EP development.

KEGG pathway analysis further substantiated that single EPs represent a distinct microbial category, characterized by differential enrichment of metabolic pathways, including aminoacyl-tRNA biosynthesis, pantothenate and CoA synthesis, pyrimidine metabolism, glycolysis/gluconeogenesis, and biosynthesis of phenylalanine, tyrosine, and tryptophan. While research on functional metabolites in the reproductive tract microbiota is limited, gut microbiota studies are more extensive. For example, intestinal microbiota produces pantothenic acid, a CoA precursor with anti-inflammatory and antioxidant properties. Gut dysbiosis can reduce pantothenic acid production, impair the citric acid cycle, and limit energy supply (29). Additionally, purine and pyrimidine metabolism provides nucleic acids for bacterial proliferation (30), and reduced aminoacyl-tRNA biosynthesis has been linked to lower abundance and diversity of enterobacteria (31). Inhibition of aminoacyl-tRNA synthesis impedes bacterial protein synthesis, representing a potential antibacterial mechanism. Collectively, such metabolic pathway alterations may contribute to endometrial pathology by disturbing vaginal and cervical microecology.

Using rank-sum tests and LDA, we identified group-specific microbial signatures. In single EP cases, *P. multocida*, *Corynebacterium striatum*, *L. iners*, and *S. amnii* were characteristic vaginal species compared with controls. Both *L. iners* and *S. amnii* were negatively correlated with reproductive tract health. The vaginal microbiome dominated by *L. iners* was associated with microecological imbalance, reduced microbiome stability, and increased vaginitis risk. Unlike other *Lactobacillus* species, *L. iners* produces only L-lactic acid and cannot synthesize D-lactic acid, which is crucial for defense against pathogenic colonization (27). The Human Microbiome Project found elevated *S. amnii* levels in the vagina of women with preterm birth (32). *S. amnii* was also positively correlated with bacterial vaginosis (BV) symptoms (33) and was a biomarker of dysbiosis (34). In the vagina and cervix, *S. amnii* was characteristic of single EPs, suggesting a role in their pathogenesis. In multiple EPs, *S. sanguinegens* and *Peptostreptococcus anaerobius* were identified as characteristic species in the vagina and cervix, respectively. *S. sanguinegens* in the cervix was associated with spontaneous abortion in Korean women (35), was more prevalent in the vagina of Indian women with preterm deliveries (36), and was associated with BV symptoms (33, 37). The relative abundance of *Peptostreptococcus anaerobius* was significantly higher in group B *Streptococcus* (GBS)-positive pregnant women (38) and in women with high-grade cervical intraepithelial neoplasia (39). These findings suggest specific microbial taxa may distinguish EP subtypes from healthy states.

Microbial differences between single and multiple EPs were notably fewer than those between EP cases and controls, suggesting that EP subtype distinctions are less pronounced than those between EP and healthy states. Compared with multiple EPs, single EP cases were enriched in vaginal *S. pneumoniae*, *A. baumannii*, *S. enterica*, and *P. multocida,* as well as cervical *P. multocida* and *A. baumannii. P. multocida* is the main causative agent for *Pasteurella* infection in humans, who usually present with symptoms, such as cellulitis and hemorrhagic drainage, from wounds (40). *S. pneumoniae* (41), *A. baumannii* (42), and *S. enterica* (43) are common infectious agents triggering inflammatory responses. Studies have reported that vaginal microorganisms could ascend to the uterine cavity, colonize, and proliferate, causing inflammation (44). Inflammation-causing microorganisms in the vagina and cervix may be linked to disease progression. However, limited evidence is available regarding the uterine cavity microbiome, and the causal relationship requires further investigation.

*L. crispatus* was characteristic of the vagina in multiple EPs. Being a vaginal predominant species in healthy individuals, *L. crispatus* is known for its positive effects on reproductive health. When the vaginal microbiome is dominated by *L. crispatus,* women have higher assisted reproduction success rates (45). Randomized controlled double-blind trials have demonstrated the effectiveness of orally or vaginally administered *L. crispatus* in alleviating the symptoms of both BV and vulvovaginal candidiasis (46). The presence of *L. crispatus* in the cervix of multiple EP cases, as opposed to single EP cases, suggests its wider distribution and abundance across the genital tract, supporting the concept of microbial continuity. Overall, the significantly higher abundance of *L. crispatus*, which favors reproductive tract microecological homeostasis, in the cervix of multiple EPs compared with single EPs may be more favorable for health in the former.

Our study highlights that the vaginal microbiota is sensitive and specific in distinguishing between single and multiple EPs. Specifically, *L. crispatus*, *Propionibacterium* sp., *A. baumannii,* and *E. faecium* are significant contributors in our predictive model. *Propionibacterium* has been recognized as an oral bacterial marker for cervical cancer (47) and shows differences between women with normal and reduced ovarian function (48). It is also more frequently isolated from the vaginas of women infected with *Chlamydia trachomatis* (49). *E. faecium* produces bioactive compounds with anticancer properties, which can directly affect cancer cell viability and cause apoptosis (50). An *E. faecium* strain found in the vagina produces enterocin, thereby inhibiting multidrug-resistant Gram-negative pathogens, including *Salmonella enterica* and *Escherichia coli*, as well as the Gram-positive pathogen *Listeria monocytogenes*, without affecting different gut lactobacilli (51). Additionally, *E. faecium* ST88Ch secretes bacteriocin-like compounds that are effective against *Candida albicans*, which causes vaginosis (52). Nevertheless, additional evidence is required to establish the relationship between these bacteria and EP-related inflammation.

Beyond taxonomic differences, our results suggest that a microbiome-based classifier could aid clinicians in distinguishing single EPs from multiple EPs, potentially guiding the extent of hysteroscopic resection or post-operative surveillance. For example, patients predicted to have multiple EPs might benefit from more aggressive follow-up or adjuvant progesterone therapy. Future research should examine whether integrating microbial biomarkers with existing clinical variables (e.g., ultrasound findings, hormonal profiles) can improve predictive accuracy in prospective settings. Additionally, these results clarify our understanding of how discrete microbial shifts translate into distinct clinical phenotypes and offer a rational basis for targeted probiotic or antimicrobial interventions.

### Limitations

Hormonal levels are known to influence the composition of the reproductive tract microbiome, with the vaginal microbiome exhibiting cyclical changes throughout the menstrual cycle (53). Hormones may also contribute to EP pathogenesis (54). Therefore, it remains unclear whether the microbiome alterations observed in EP are directly mediated by hormones. To minimize confounding factors, future studies should consider assessing patients' hormonal levels during sample collection.

While our sample size was adequate for a pilot study, it may limit statistical power and generalizability. These findings should be considered preliminary and validated in larger, multi-center cohorts. Furthermore, as our study exclusively enrolled Asian women, the results may not be generalizable to other ethnic groups. Jacques Ravel et al. documented significant racial differences in CST proportions among asymptomatic women of diverse ethnicities (55), indicating that ethnicity influences microbial composition in healthy women. For instance, Black women’s microbiomes tend to exhibit higher alpha diversity, greater abundance of *L. iners*, and lower abundance of *L. crispatus* (56). In disease states, microbiome variations across racial groups are also evident. For example, in the context of HPV carcinogenicity, race modifies the protective effect of an optimal vaginal microbiome, with non-Hispanic Black women deriving less benefit than non-Hispanic White women (57). Collectively, these findings suggest that ethnicity significantly shapes the microbiota in both health and disease states. Further validation is therefore necessary to determine whether our conclusions can be extrapolated to other populations.

### Conclusion

In summary, the characteristic species associated with single EPs are predominantly inflammation-associated bacteria, suggesting potential pathogenicity. By contrast, the characteristic species linked to multiple EPs are largely involved in maintaining reproductive tract homeostasis. Our study demonstrates that EP subtypes exhibit distinct microbial patterns, which may be clinically useful for subtype identification. Given our modest sample size, future prospective studies with larger and more diverse patient populations are warranted to confirm the diagnostic potential of these identified microbial signatures. Future research should explore how these differential species contribute to EP development, thereby enhancing our understanding of EP pathogenesis.

## Data Availability

The raw data and the STORM checklist were deposited into the CNGB Sequence Archive (CNSA) of China National GeneBank DataBase (CNGBdb, https://db.cngb.org/; accession number: CNP0006762).

## SUPPLEMENTAL MATERIAL

Figure S1 msystems.00023-25-s0001.docx Relative abundance of key differential species.

Table S1 msystems.00023-25-s0002.csv β-Diversity in the vagina and cervix of the control, single EP, and multiple EP groups.

Table S2-1 msystems.00023-25-s0003.csv Relative abundance of species in vaginal samples.

Table S2-2 msystems.00023-25-s0004.csv Relative abundance of species in cervical samples.

Table S3-1 msystems.00023-25-s0005.csv KEGG pathways associated with single EP and multiple EP in vaginal samples.

Table S3-2 msystems.00023-25-s0006.csv KEGG pathways associated with single EP and multiple EP in cervical samples.

Table S4-1 msystems.00023-25-s0007.csv Differential species between the single EP and control group.

Table S4-2 msystems.00023-25-s0008.csv Differential species between the multiple EP and control group.

Table S4-3 msystems.00023-25-s0009.csv Differential species between the single EP and multiple EP group.

Table S5 msystems.00023-25-s0010.csv Essential clinical parameters incorporated into a first random forest analysis.

ASM does not own the copyrights to Supplemental Material that may be linked to, or accessed through, an article. The authors have granted ASM a non-exclusive, world-wide license to publish the Supplemental Material files. Please contact the corresponding author directly for reuse.
