# Supplementary material for: Exploring cervicovaginal microbiome differences between single and multiple endometrial polyps: implications for non-invasive classification
Source: mSystems. 2025 Sep 15;10(10):e00023-25. doi: 10.1128/msystems.00023-25 (PMC12542703; doi:10.1128/msystems.00023-25)
Supplement: Figure S1 — Relative abundance of key differential species. [file msystems.00023-25-s0001.docx]

**
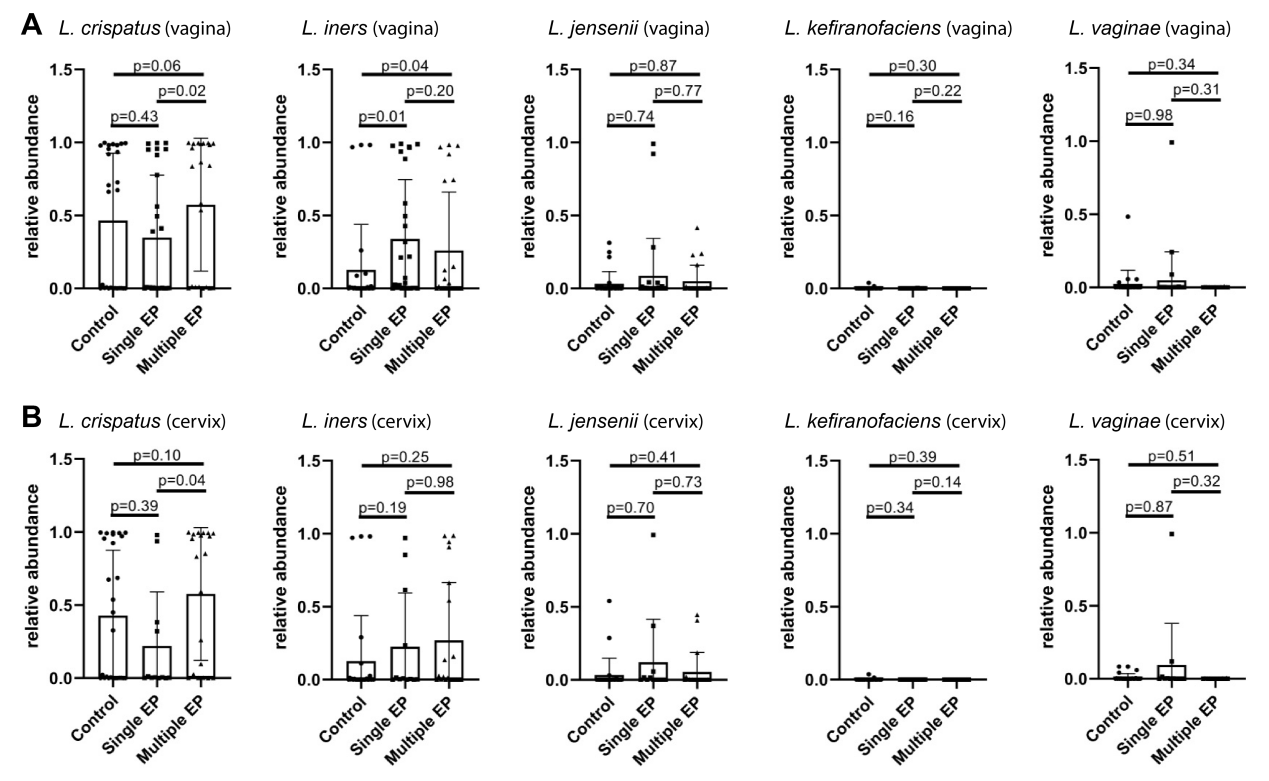
Figure S1. The relative abundance of key differential species among healthy, single EP and multiple EP.**

1. Scatterplot of relative abundance of key differential species in the vagina, with Wilcoxon p value. B) Scatterplot of relative abundance of key differential species in the cervix, with Wilcoxon p value.
